# Reviewed article: Batista JFC, Santos MAA, Lima SO. Trend of epidemiological indicators and factors associated with tuberculosis treatment dropout and death among street people in Brazil: an ecological and cross-sectional study, 2014-2022. Epidemiol Serv Saude. 2025:34;20240273

**DOI:** 10.1590/S2237-96222024v34e20240273.a

**Published:** 2025-04-14

**Authors:** Gabriel Pavinati

**Affiliations:** Universidade Estadual de Maringá, Departamento de Enfermagem

**Table:** 

Rating	Excellent	Good	Average	Below average	Poor
Interest	✓				
Quality			✓		
Originality		✓			
Overall		✓			

**Table:** 

Questionnaire	Yes	No	Not applicable
Does the manuscript contain new and significant information to justify publication?	✓		
Does the Abstract (Summary) clearly and accurately describe the content of the article?		✓	
Is the problem significant and concisely stated?		✓	
Are the methods described comprehensively?		✓	
Are the interpretations and conclusions justified by the results?	✓		
Is adequate reference made to other work in the field?		✓	

## Manuscript Structure:

Length of article is: Adequate

Number of tables is: Adequate

Number of figures is: Adequate

Please state any conflict(s) of interest that you have in relation to the review of this paper (state “none” if this is not applicable): None

## Comments

Introdução

No terceiro parágrafo os autores repetem a ideia do primeiro parágrafo da mesma maneira: “Pessoas em situação de rua são consideradas pelo Ministério da Saúde como grupo de vulnerável à tuberculose”. Sugiro reescreve-la para não ficar tão repetitivo.

Ao término da introdução os autores destacam: “Há uma escassez de estudos sobre a temática (8), principalmente voltados aos fatores associados aos desfechos da tuberculose em pessoas em situação de rua. Na literatura, há somente estudo brasileiro de representação estadual (9)”. No entanto, ao aplicar no buscador de referência os termos “tuberculose”, “fatores associados” e “pessoa em situação de rua” encontrei ao menos mais um estudo, esse de âmbito nacional: Pavinati, G., Lima, L. V. D., Teixeira, C. S. S., Hino, P., Bertolozzi, M. R., Nery, J. S., & Magnabosco, G. T. (2024). Vulnerabilidade à perda de seguimento e ao óbito por tuberculose nas pessoas em situação de rua no Brasil: um estudo de coorte retrospectiva. Ciência & Saúde Coletiva, 29(7), e02742024. Importante considerar a sua existência, inclusive para corroborar ou refutar os achados.

Ainda na introdução, os autores justificam o estudo de fatores associados, mas não o de tendência. Destaque também a relevância da sua condução.

Método

Os autores apresentam “Estudo transversal e ecológico exploratório e analítico, com abordagem quantitativa, subdividido em análise temporal dos indicadores da tuberculose e análise dos fatores associados aos desfechos desfavoráveis em pessoas em situação de rua no Brasil”. Penso que a frase está pouco coesa. Sugiro que seja feita a reescrita. Ainda questiono: essa pesquisa é mesmo exploratória? Não seria uma pesquisa epidemiológica com dois diferentes delineamentos para condução da tendência e fatores associados?

Referenciar o RECORD

Informa-se: Foram utilizados dados públicos referentes às tuberculoses no Brasil e macrorregiões no período de janeiro de 2014 a dezembro de 2022. Sugiro deixar a palavras tuberculose no singular. Além disso, existe um artigo publicadas na Revista Epidemiologia e Serviços de Saúde, conduzida por trabalhadores do Programa Nacional de Tuberculose apontando que a inserção da variável PSR na ficha de notificação se deu apenas em 2014. Como os autores tiveram acesso a dados anteriormente a esse período? Segue artigo mencionado para conhecimento dos autores: Rocha, Marli Souza et al. Sistema de Informação de Agravos de Notificação (Sinan): principais características da notificação e da análise de dados relacionada à tuberculose. Epidemiologia e Serviços de Saúde [online]. v. 29, n. 1 [Acessado 24 Agosto 2024] , e2019017. Disponível em: <https://doi.org/10.5123/S1679-49742020000100009>. ISSN 2237-9622. https://doi.org/10.5123/S1679-49742020000100009.

Na ficha de notificação, para situação de encerramento, tem-se: óbito e óbito por outras causas. Como os autores trabalharam essas diferentes categorias? Mencionar.

Apresenta-se que: “Além disso, os óbitos não foram extraídos do Sistema de Informação sobre Mortalidade devido a impossibilidade de classificar as mortes segundo população de rua”. Acredito ser mais válido informar de onde foi extraído o dado.

A frase: “Os dados referentes aos casos confirmados de tuberculose foram extraídos do Sistema de Informação de Agravos e Notificação (Sinan), enquanto a estimativa da população em situação de rua foi proveniente do Instituto de Pesquisa Econômica

Aplicada”, foi apresentada no parágrafo anterior. Rever.

Em um momento os autores apresentam que para os cálculos foram considerados “casos novos ou óbitos por tuberculose em pessoas em situação de rua em determinado local e período”; depois, afirma-se que foram consideradas variáveis clínicas “tipo de entrada - caso novo, recidiva, reingresso após abandono, pós-óbito, não sabe”; mais a frente diz-se: “tipo de entrada reingresso pós-abandono e recidiva foram aglutinados no grupo retratamento. Talvez essa seção precisa ser revista trazer maior clareza aos passos metodológicos. Os dados foram triados de duas maneiras distintas para cada análise?

Considerando o grande quantitativo de dados ignorados em relação à notificação dos casos de tuberculose em pessoas em situação de rua, normalmente, superior à 20%; como esse fenômeno interfere na imputação?

Sobre o tipo de estudo, os autores o conceituaram como transversal. No entanto, pensando na natureza da coleta do dados, esse foi coletado ao longo de todo o tratamento de cada paciente: do diagnóstico, baciloscopias de acompanhamento e desfecho. Portanto, seguindo a premissa dos estudos epidemiológicos, essa pesquisa seria melhor conceituada como coorte retrospectiva. Apesar de os autores fazerem a coleta de dados em um único momento na base de dados, a origem do dados é longitudinal pois temos o seguimento do paciente.

As razões de chances ajustadas foram ajustados ao que?

Acredito que a tabela suplementar 1 precisa ser revista: os dados faltantes relativos ao TDO são superiores a cinco mil casos, dos 21 mil notificados. Os autores colocam que isso representa 2,4% do total, quando na verdade isso é superior a 20%. Para expressar adequadamente os dados faltantes, comumente se apresenta o número de informações ignoradas pelo total de casos notificados, isso para cada variável individualmente. Como os autores conduziram?

Descrever mais detalhadamente como foi construído o indicador de acompanhamento.

Na figura 1, primeiro apresentar incidência, antes das variáveis de desfecho, seguindo a lógica de ocorrência dos eventos.

Recomendo inverter a apresentação das tabelas 1 e 2. Deve-se seguir a lógica de apresentação em que primeiro se apresenta os dados descritivos e depois os analíticos. Portanto, transforma-se a tabela 2 em 1; e a tabela 1 em 2.

Discussão

A discussão apresenta parágrafos que carecem de referências, por exemplo: “com indicadores epidemiológicos longe das metas estabelecidas pela organização mundial da saúde, de no mínimo 85% de cura, máximo de 5% de abandono e 1 morte por 100 mil habitante”. Referenciar OMS.

A discussão está confusa. Apresentam-se todos os achados novamente de maneira conjunta. Sugiro trazer os achados relevantes na sequência em que são apresentados no resultado, já contrastando com a literatura e levantando hipóteses ou recomendações.

No seguinte parágrafo: “As taxas de incidência, mortalidade, a proporção de cura e abandono de tratamento em pessoas em situação de rua identificados na presente pesquisa, são desfavoráveis e discrepantes quando comparados a outros grupos vulneráveis. Um estudo com pessoas privadas de liberdade, no período de 2007-2013 apontou uma proporção de cura de 68,6%, abandono de 10,7%, incidência de 852,80/100 mil e mortalidade de 15,70/100 mil (15), enquanto uma pesquisa em indígenas (2011-2017) a incidência média foi de 109,0/100 mil (16)”. Os autores comparam os dados da PSR com outros grupos vulneráveis. Acho que esse caminho não é interessante para discussão. Cada grupo vulnerável tem a sua particularidade e não há mérito em ser vulnerável. Por exemplo: a pessoa privada de liberdade é vulnerável, mas consegue atingir maior cura pois está cerceada em um espaço físico e, em geral, o tratamento é acompanho de perto pelo serviço prisional; ao passo que a PSR é itinerante, com pouco ou nenhum vínculo com serviços de saúde e assistência social, o que dificulta o seguimento. Sugiro que as comparações sejam feitas com a população que não está em situação de rua, pois a raiz do problema é esse.

Qual a justificativa levantada pelos autores acerca da seguinte frase: “Além disso, a incidência em pessoas em situação de rua foi superior a países como Portugal (2008-2010) (122/100 mil) (17) e Estados Unidos (1997-2010) (47/100 mil) (18)”? Há mais casos ou há mais diagnóstico?

Na frase: “Foi verificado nesse estudo que a taxa de incidência e mortalidade apresentaram redução anual somente no Sudeste. Evidência contrária foi identificada por Scholze e colaboradores (22) em pessoas em situação de rua do Paraná, onde no período de janeiro de 2014 a dezembro de 2018, a incidência de tuberculose cresceu 38,3% (IC95%: 23,3; 55,2) ao mês”. Não observei contrariedade entre os estudos. Acho que a frase precisa ser revista.

“Dessa forma, essa diferença pode ser atribuída à pandemia da COVID-19.” Frase truncada. Precisa rever.

Na frase: “além de erros na classificação da raça/cor da pele, por ser autodeclarada”. Não compreendi, como a autodeclaração pode ser frágil?

Em relação ao banco de dados e a lista de codificação utilizados na pesquisa, o link informado não permitiu acesso.

Sugiro incluir na discussão aspectos relacionados à política de proteção social destinado aos pacientes de tuberculose e como a PSR pode se beneficiar disso; também recomendo citar a existência de uma política nacional de cuidado à PSR e problematizar a sua real implementação.

Referências

Contei ao menos 18 referências anteriores a cinco anos. Considerando que isso representa mais de 50% do total, recomendo fortemente substituir aquelas não essenciais por literatura mais recente.

Rever resumo e descritores de acordo com as mudanças sugeridas acima. 

